# Polyglycerol-functionalized nanodiamond as a platform for gene delivery: Derivatization, characterization, and hybridization with DNA

**DOI:** 10.3762/bjoc.10.64

**Published:** 2014-03-24

**Authors:** Li Zhao, Yuki Nakae, Hongmei Qin, Tadamasa Ito, Takahide Kimura, Hideto Kojima, Lawrence Chan, Naoki Komatsu

**Affiliations:** 1 Department of Chemistry, Shiga University of Medical Science, Seta, Otsu 520-2192, Japan; 2 Department of Stem Cell Biology and Regenerative Medicine, Shiga University of Medical Science, Seta, Otsu 520-2192, Japan; 3 Department of Materials Science and Engineering, Faculty of Science and Technology, Meijo University, Tempaku, Nagoya 468-8502, Japan; 4 Departments of Medicine, and Molecular and Cellular Biology, Baylor College of Medicine, Houston, Texas 77030, USA

**Keywords:** carbon-nanomaterials, click chemistry, DNA, gene delivery, nanodiamond, polyglycerol, polypeptides

## Abstract

A gene vector consisting of nanodiamond, polyglycerol, and basic polypeptide (ND-PG-BPP) has been designed, synthesized, and characterized. The ND-PG-BPP was synthesized by PG functionalization of ND through ring-opening polymerization of glycidol on the ND surface, multistep organic transformations (–OH → –OTs (tosylate) → –N_3_) in the PG layer, and click conjugation of the basic polypeptides (Arg_8_, Lys_8_ or His_8_) terminated with propargyl glycine. The ND-PG-BPP exhibited good dispersibility in water (>1.0 mg/mL) and positive zeta potential ranging from +14.2 mV to +44.1 mV at neutral pH in Milli-Q water. It was confirmed by gel retardation assay that ND-PG-Arg_8_ and ND-PG-Lys_8_ with higher zeta potential hybridized with plasmid DNA (pDNA) through electrostatic attraction, making them promising as nonviral vectors for gene delivery.

## Introduction

A variety of nanoparticles have been investigated as nonviral vectors in drug and gene delivery systems [1–2]. Among these nanoparticles, nanodiamond (ND) has attracted a great deal of attention due to its high chemical stability, low toxicity, and large specific surface area [3–6]. In addition, various functions can be added to ND through organic reactions on the ND surface [4–5,7–11]. In this sense, ND has an advantage over non-carbonaceous nanomaterials, because the methodology in synthetic organic chemistry can be applied to the ND surface, which is covered with organic functional groups [12]. Quite recently, we found that ring-opening polymerization of glycidol is initiated at the oxygen-containing functionalities, hydroxy and carboxy groups, on the ND surface to give polyglycerol- (PG) grafted ND with 30 nm size (ND30-PG) [13]. The resulting ND30-PG exhibited very good dispersibility not only in water (>20 mg/mL), but also in phosphate buffererd saline (PBS) (>16 mg/mL), making the in vivo use of ND more promising in biomedical applications. In addition, the good dispersibility and a large number of hydroxy groups of ND-PG enable further surface functionalization to add more functions to ND [14].

As for gene delivery, on the other hand, DNA was immobilized on the surface of nanoparticles mostly by electrostatic attraction between the negative charge of DNA and the positive charge on the surface of the nanoparticle [15]. In the case of ND, for example, basic polypeptides [16], polyamine polymer [17], primary and tertiary amines [17–18], and quaternary ammonium salts [19] were employed to coat ND covalently or noncovalently as positively charged ligands for DNA immobilization. Although the functionalized ND is proven to immobilize DNA, more functions such as enough dispersibility and targeting efficacy are required to use ND in vivo as a gene vector. Therefore, a more reliable and general process is desired to add sufficient functions for ND-based gene vectors. Herein a conjugation of ND-PG with basic polypeptides (Arg_8_, Lys_8_ and His_8_) through click chemistry followed by hybridization with plasmid DNA (pDNA) and its characterization by electrophoresis is reported.

## Results and Discussion

### Preparation and characterization of ND50-PG

**Figure 1:**
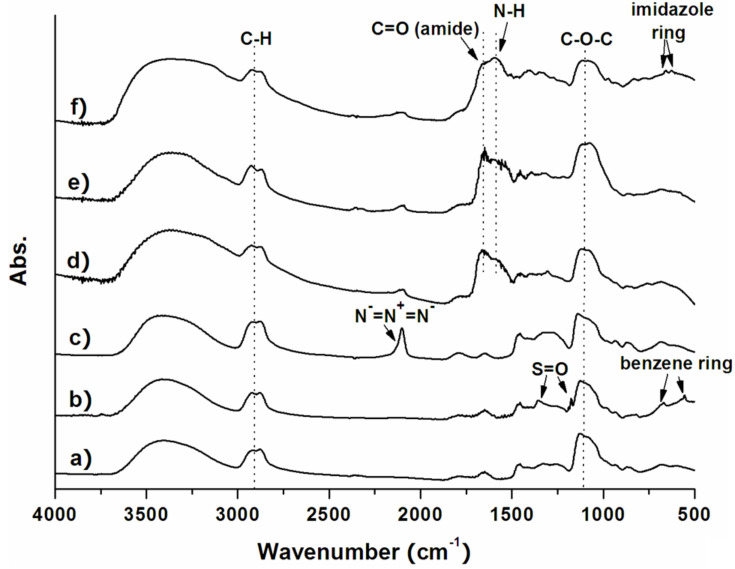
FTIR spectra of a) ND50-PG, b) ND-PG-OTs, c) ND-PG-N_3_, d) ND-PG-Arg_8_, e) ND-PG-Lys_8_, and f) ND-PG-His_8_. Arrows indicate new absorption bands in each step.

**Figure 2:**
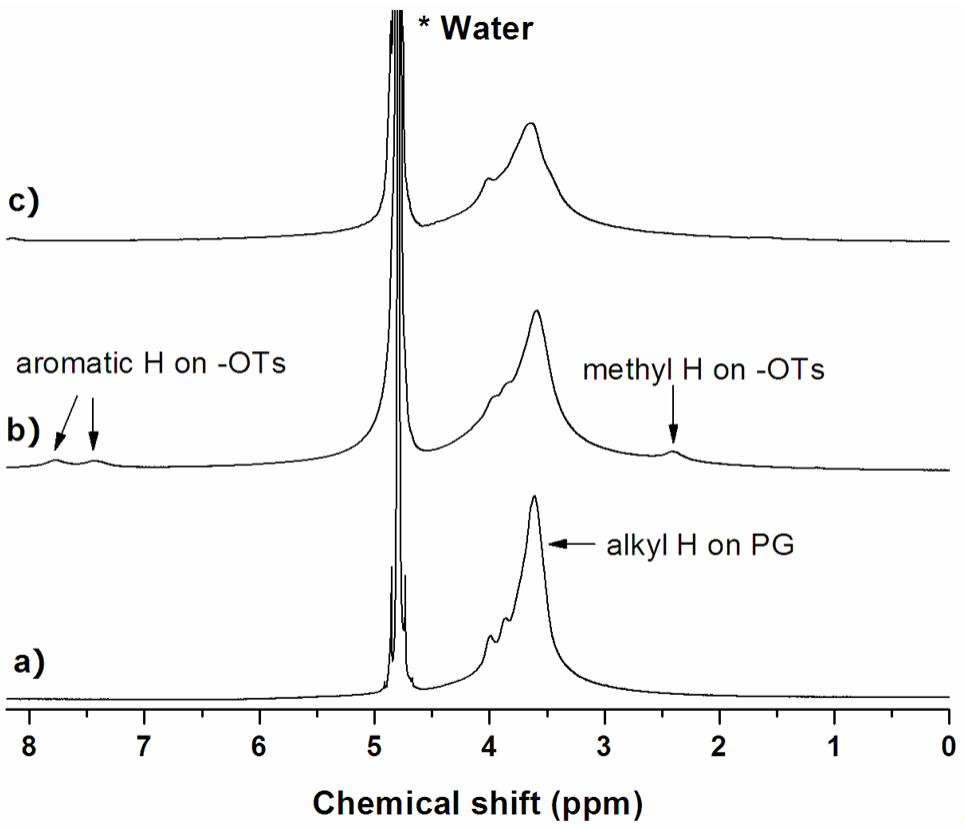
^1^H NMR spectra of a) ND50-PG, b) ND-PG-OTs and c) ND-PG-N_3_ in D_2_O.

**Figure 3:**
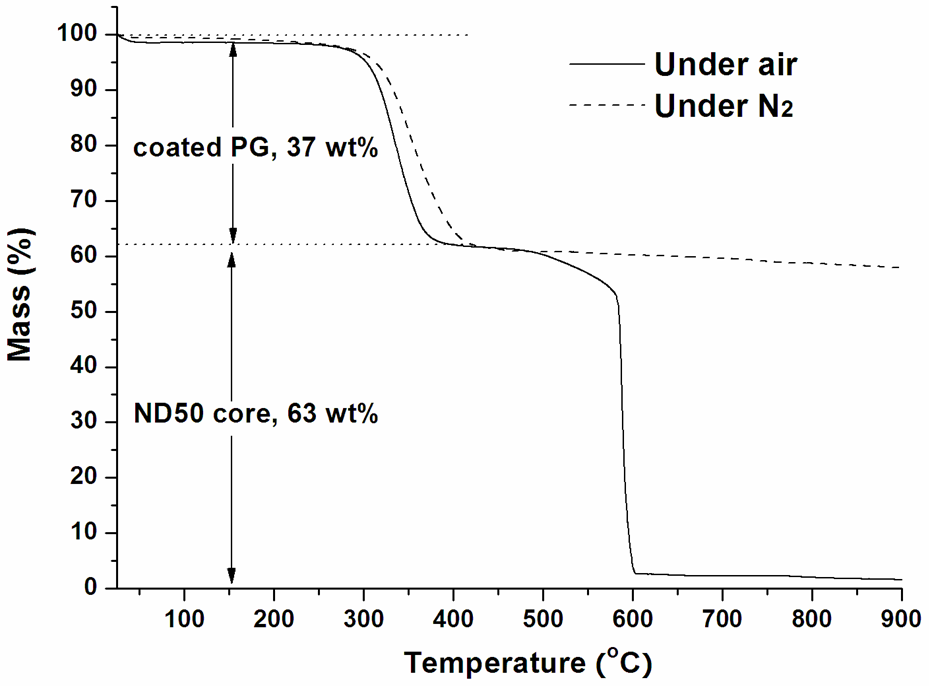
TGA profiles of ND50-PG under nitrogen and air.

In view of actual cancer therapy utilizing the enhanced permeation and retention (EPR) effect, ND with 50 nm size was chosen for this study. The ND50 was covalently functionalized with hyperbranched PG through ring-opening polymerization of glycidol according to the procedure we reported previously [13]. The resulting ND50-PG was characterized qualitatively by FTIR and ^1^H NMR, and quantitatively by TGA. The IR (Figure 1a) and NMR spectra (Figure 2a) of ND50-PG are almost the same as those of ND30-PG [13], proving the PG grafting on the ND50. In addition, PG:ND weight ratio of ND50-PG (37:63) is almost the same as that of ND30-PG (40:60) in TGA (Figure 3), though ND50 has a smaller specific surface area than ND30. Accordingly, the dispersibility (>20 mg/mL) of ND50-PG in water is almost the same as that of ND30-PG [13]. However, ND5-PG showed an opposite weight ratio (PG:ND = 78:22), though IR and NMR spectra were almost the same as those of ND30-PG and ND50-PG [20]. The much larger weight ratio of the PG layer improved the dispersibility of ND5-PG significantly (>80 mg/mL in PBS), implying that the dispersibility of ND-PG is proportional to the weight ratio of PG to the ND core.

**Figure 4:**
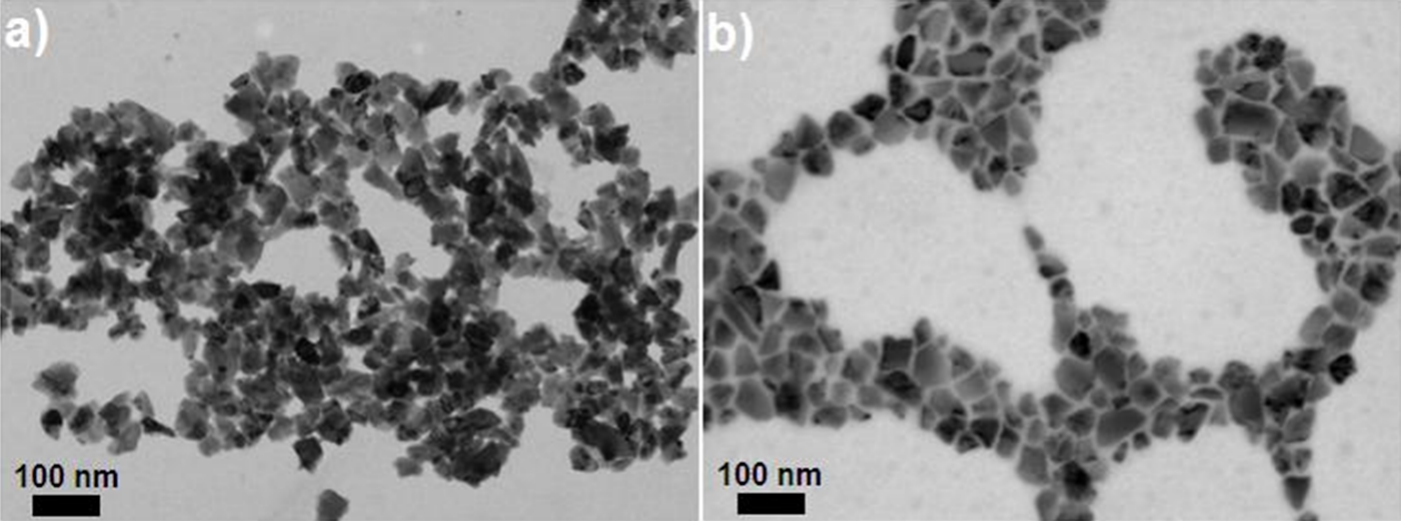
STEM images of a) pristine ND50 and b) ND50-PG.

The particle size of ND50 and ND50-PG was measured by STEM and DLS. In contrast to bare ND50 particles that are prone to aggregate (Figure 4a), ND50-PG is individually dispersed as shown in Figure 4b. Moreover, 5–10 nm blank space between the ND particles in the STEM image (Figure 4b) may be attributed to the PG layer on the ND surfaces. The average core diameter and the mean hydrodynamic diameter of ND50-PG were determined to be 52.2 ± 14.4 nm by STEM (Figure 4b) and 66.9 ± 14.8 nm by DLS (Table 1), respectively. Based on the difference of the core and hydrodynamic diameters, the thickness of the PG layer on the ND50-PG was estimated to be ca. 7 nm, which is in agreement with the inter-particle distance in the STEM image (Figure 4b) as mentioned above.

**Table 1:** Hydrodynamic diameter and zeta potential of nanoparticles in Milli-Q water.

Nanoparticle	Hydrodynamic diameter^a^ (nm)	Zeta potential (mV)

ND50	52.8 ± 20.3	–46.7 ± 3.5
ND50-PG	66.9 ± 14.8	–36.8 ± 1.7
ND-PG-Arg_8_	372 ± 105	+44.1 ± 1.9
ND-PG-Lys_8_	176 ± 44	+38.7 ± 1.4
ND-PG-His_8_	195 ± 64	+14.2 ± 0.5

^a^Mean diameter ± SD was determined by DLS on the basis of number distribution.

### Immobilization of basic polypeptides through click chemistry

**Scheme 1:**
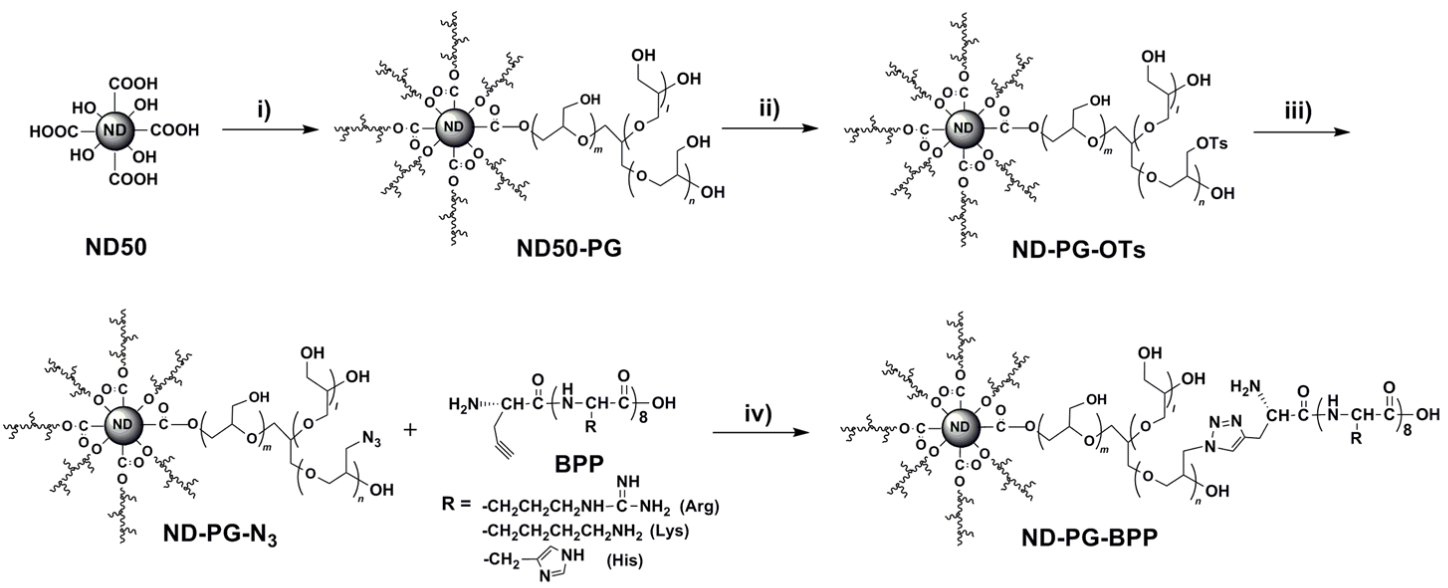
Synthetic route from ND50 to ND-PG-BPP; i) glycidol, 140 °C, 20 h; ii) *p*-TsCl, pyridine, 0 °C to rt; iii) NaN_3_, 90 °C, overnight; iv) copper(II) sulfate pentahydrate, sodium ascorbate, rt, 96 h.

**Figure 5:**
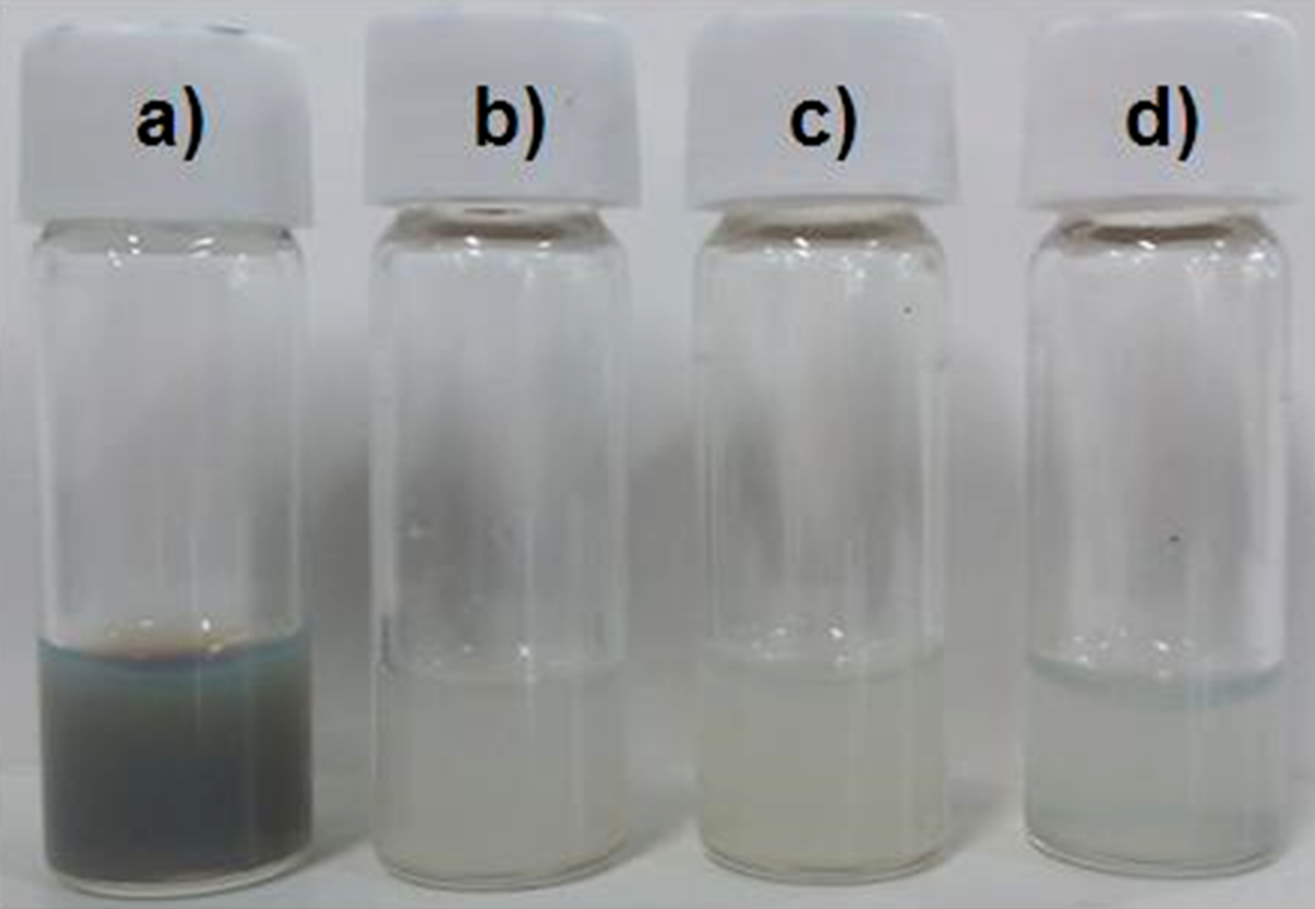
Picture of the dispersions of a) ND50-PG (20 mg/mL), b) ND-PG-Arg_8_, c) ND-PG-Lys_8_ and d) ND-PG-His_8_ (b–d, 1.0 mg/mL) in water.

The PG layer including a large number of hydroxy groups endows ND50-PG not only with very high hydrophilicity (Figure 5), but also with a versatile platform for further surface engineering. The synthetic route from ND50-PG to ND-PG-BPP is shown in Scheme 1. Some of the hydroxy groups of ND50-PG were reacted with tosyl chloride (TsCl) in pyridine and the resulting tosylates (ND-PG-OTs) were substituted by azide (ND-PG-N_3_). The azido groups reacted with the alkyne group at the end of the polypeptides (click chemistry) to produce ND-PG-BPP [12,21–22].

A series of reactions from ND-PG to ND-PG-BPP were monitored by FTIR (Figure 1) [20]. The absorption bands at 1350 cm^−1^ and 1176 cm^−1^ are attributed to asymmetric and symmetric stretchings of S→O bonds of the tosyl group in ND-PG-OTs, respectively. Another two new absorption bands at 556 and 669 cm^−1^ are assigned to the bending vibrations of aromatic C–H (Figure 1b). The ND-PG-N_3_ clearly shows a characteristic strong absorption band at 2100 cm^−1^ corresponding to the azido group (Figure 1c). The azido absorption band disappeared after the click conjugation of the BPP (Figure 1d–f), indicating the complete consumption of azido groups. The immobilization of polypeptides was verified by the absorption bands at 1650 and 1590 cm^−1^, which correspond to the C=O stretching and N–H bending of amide bonds in the polypeptide. In the case of ND-PG-His_8_, absorption peaks at 624 and 657 cm^−1^ are attributed to C–H bending of the imidazole rings in the polyhistidine (Figure 1f).

Taking advantage of the good dispersibility of the ND50-PG and their derivatives, they are characterized by solution-phase ^1^H NMR (Figure 2). As shown in Figure 2b, the peaks at 7.7 and 7.4 ppm are assigned to the aromatic hydrogens of the tosyl group, and the methyl hydrogens are found at 2.3 ppm. These three peaks of the ND-PG-OTs in Figure 2b disappeared in Figure 2c after the reaction with sodium azide, indicating the complete substitution of the tosyl group.

The hydrodynamic diameter of ND-PG-BPP in water largely increased to more than 150 nm (Table 1), indicating that aggregation occurred in the dispersions. Since ND-PG-BPP has positive charge as will be discussed below, the charges may be connected by some anions to assemble the particles. However, ND-PG-BPP still has dispersibility of >1.0 mg/mL with less stability (Figure 5).

### Zeta potential characterization and pDNA complexation

To analyze the surface charge of the nanoparticles, we measured the zeta potential of ND50, ND50-PG and ND-PG-BPP at neutral pH in Milli-Q water. The results are summarized in Table 1. ND50 showed a relatively high negative zeta potential of –46.7 ± 3.5 mV because of a large number of carboxylic groups on the surface. The zeta potential changed to –36.8 ± 1.7 mV by PG coating of ND50, probably because some of the carboxylic groups (protic functional groups) are converted to ester (aprotic ones) by initiation of the ring-opening polymerization of glycidol. The immobilization of polypeptides turned the zeta potentials into plus (–36.8 mV → +14.2 to +44.1 mV) due to the protonation to the basic groups in the peptides; imidazole, amine, and guanidine. These zeta potentials of the ND-PG-BPP are roughly propotional to the p*K*a values of the side chains in these basic amino acids; His (6.0), Lys (10.5), and Arg (12.5).

**Figure 6:**
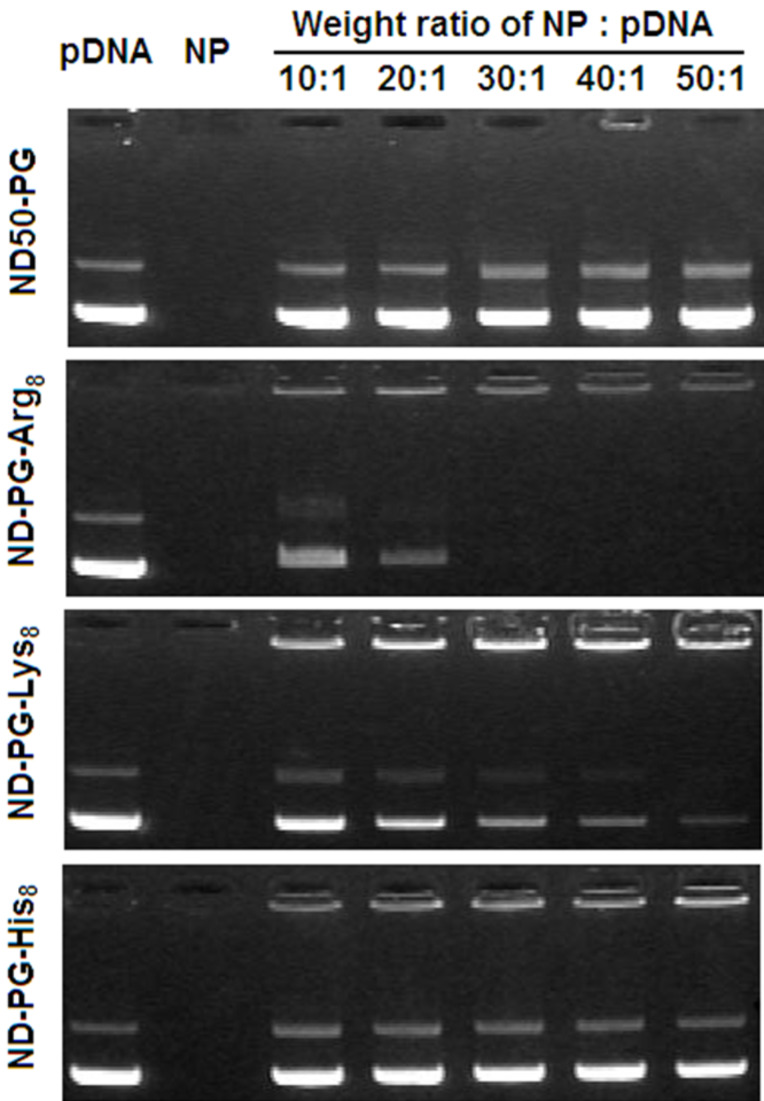
Electrophoretic migration of pDNA, NP (ND50-PG or ND-PG-BPP), and NP/pDNA mixtures at various weight ratios.

The positive surface charge of nanoparticles enables complexation with negatively charged DNA through electrostatic interaction. To analyze the DNA complexation capability of the ND-PG-BPP, we performed an agarose gel retardation assay. The result of the electrophoresis is shown in Figure 6. ND-PG-Arg_8_ and ND-PG-Lys_8_ with higher positive zeta potential formed complexes with the pDNA, which can be seen as becoming light of density of the pDNA bands. In particular, ND-PG-Arg_8_ with the highest positive zeta potential completely retarded the pDNA at a low NP:pDNA weight ratio (30:1). In contrast, ND50-PG and ND-PG-His_8_ were not able to form a complex with the pDNA even at the highest NP:pDNA weight ratio (50:1).

## Conclusion

We have prepared ND-PG-BPP through multistep organic transformations including click chemistry. The PG layer on ND gave good aqueous dispersibility, enabling derivatization and characterization in the solution phase. The ND-PG-Arg_8_ and ND-PG-Lys_8_ possessing relatively high positive zeta potential immobilized the pDNA, demonstrating their potential of ND-PG-BPP as vectors for gene delivery.

## Experimental

### Materials and instruments

All the reagents and solvents used for the synthesis were employed as received. ND with 50 nm median diameter (ND50), prepared from high-pressure-high-temperature (HPHT) bulk diamond, was kindly provided by Tomei Diamond Co., Ltd. (Lot. No. 66093). Glycidol was purchased from Kanto Chemical Co., Ltd. *p*-Toluenesulfonyl chloride and sodium azide were purchased from Nacalai Tesque, Co. Basic polypeptides binding propargyl glycine (G*) at an N terminal (G*BPP) were obtained from two sources; G*Lys_8_ and G*His_8_ were synthesized by the central research laboratory of Shiga University of Medical Science and G*Arg_8_ was purchased from GL Biochem Ltd. in Shanghai, China. The pBluescript II KS (Agilent Technologies, Inc., Tokyo, Japan) was used as a test pDNA for the hybridization with ND-PG-BPP. Dialysis was carried out by use of Spectra/Pro^®^ dialysis membrane, MWCO: 12–14 kDa.

FTIR spectral measurements were conducted using IR Prestige-21 (Shimadzu Co.). Samples were prepared by drop-coating of suspension to form a thin film on a stainless alloy plate, and then dried at 70 °C. Hydrodynamic diameters in solution were determined by dynamic light scattering (DLS) using a Nanotrac UPA-UT151 system (Microtrac, Inc.). ^1^H NMR spectra (270 MHz) were recorded on a JEOL Model JNM-EX270 spectrometer. Scanning transmission electron microscopy (STEM) was performed on a JEOL JSM-7500F field emission scanning electron microscope at 25 kV accelerating voltage for the TEM model. All samples for electron microscopy were prepared by evaporating one drop (~50 µL) of samples on ultrathin carbon-coated copper grids. Thermogravimetric analyses (TGA) were carried out by a Q-50 analyzer (TA instruments) with a heating rate of 20 °C/min under a nitrogen or an air flow (60 mL/min). Zeta potential measurement was conducted in water solutions using an Otsuka ELSZ-1 zeta-potential analyzer.

### Synthesis and characterization

#### ND50-PG

ND50-PG was prepared according to our reported method [13] using ND50 as a starting material, and characterized by FTIR (Figure 1a) and solution-phase ^1^H NMR (Figure 2a).

#### ND-PG-OTs

ND50-PG (100 mg) was dissolved in pyridine (4.0 mL) by bath sonication and then cooled down to 0 °C in an ice/water bath. *p*-Toluenesulfonyl chloride (200 mg, 1.05 mmol) was dissolved in pyridine (2.0 mL) and added dropwise into the mixture under rapid stirring. The solution was stirred at 0–5 °C for 3 h and at room temperature overnight. The resulting solid was collected by centrifugation (Beckman Coulter Avanti^®^ J-E centrifuge) at 50400 *g* and purified in DMF by repeated redispersion/centrifugation cycles. It was characterized by FTIR (Figure 1b) and solution-phase ^1^H NMR (Figure 2b).

#### ND-PG-N_3_

In a similar manner to our reported method [20], sodium azide (100 mg, 1.54 mmol) in water (2.0 mL) was added into ND-PG-OTs (80 mg) in DMF (6.0 mL) and stirred at 90 °C overnight. After cooling down, the product was collected by centrifugation and purified in water by repeated redispersion/centrifugation cycles. It was characterized by FTIR (Figure 1c) and solution-phase ^1^H NMR (Figure 2c).

#### ND-PG-BPP

The click reaction of ND-PG-N_3_ and G*BPP was conducted in a similar manner to our reported procedure [22]. G*BPP (8.0 mg) was added to a solution of ND-PG-N_3_ (10 mg) in water (2.0 mL). Copper(II) sulfate pentahydrate (8.0 mg) in water (0.5 mL) and sodium ascorbate (10 mg) in water (0.5 mL) were added into the mixture with vigorous stirring. The resulting brown suspension was bath-sonicated for 10 min and then stirred at room temperature for 96 h. Diluted ammonia was dropped into the suspension to dissolve insoluble copper salts, giving a blue-gray suspension. The solid was collected by centrifugation and washed with 1% ammonia repeatedly. The washed sample was dialyzed against Milli-Q water to thoroughly remove ammonia. The resulting ND-PG-BPP were characterized by FTIR (Figure 1d–f). Decent NMR spectra of the ND-PG-BPP were not obtained because of lower dispersibility of the ND-PG-BPP than the above ND-PG derivatives.

#### Gel retardation assay

The hybridization of ND-PG-BPP with DNA was studied by means of an agarose gel retardation assay. The agarose gel was prepared by dissolving 1% (w/v) agarose in tris-acetate-EDTA (TAE) buffer containing ethidium bromide (0.1 mg/mL). The ND-PG-BPP was mixed with 0.2 μg of pDNA in 10 μL double-distilled water at designated NP (ND-PG-BPP):pDNA weight ratios (Figure 6). The mixture together with ND-PG-BPP and pDNA were loaded into the slots of the gel and subjected to electrophoresis at a voltage of 100 V for 20 min. The pDNA in the gel was visualized and photographed on a FAS-IV ultraviolet transilluminator (Nippon Genetics Co. Ltd).
